# Electronic Nudge Letters to Increase Influenza Vaccination Uptake in Younger and Middle-Aged Individuals With Diabetes

**DOI:** 10.1016/j.jacadv.2024.101391

**Published:** 2024-11-13

**Authors:** Mats C. Højbjerg Lassen, Niklas Dyrby Johansen, Muthiah Vaduganathan, Ankeet S. Bhatt, Daniel Modin, Safia Chatur, Brian L. Claggett, Kira Hyldekær Janstrup, Carsten Schade Larsen, Lykke Larsen, Lothar Wiese, Michael Dalager-Pedersen, Lars Køber, Scott D. Solomon, Pradeesh Sivapalan, Jens Ulrik Stæhr Jensen, Cyril Jean-Marie Martel, Tyra Grove Krause, Tor Biering-Sørensen

**Affiliations:** a Department of Cardiology, Copenhagen University Hospital–Herlev and Gentofte, Copenhagen, Denmark; b Center for Translational Cardiology and Pragmatic Randomized Trials, Department of Biomedical Sciences, Faculty of Health and Medical Sciences, University of Copenhagen, Copenhagen, Denmark; c Cardiovascular Division, Brigham and Women’s Hospital, Harvard Medical School, Boston, Massachusetts, USA; d Center for Cardiometabolic Implementation Science, Brigham and Women’s Hospital, Boston, Massachusetts, USA; e Kaiser Permanente San Francisco Medical Center & Division of Research, San Francisco, California, USA; f Stanford University School of Medicine, Palo Alto, California, USA; g Department of Clinical Medicine–Department of Infectious Diseases, Aarhus University Hospital, Aarhus, Denmark; h Research Unit for Infectious Diseases, Odense University Hospital, Odense, Denmark; i Department of Infectious Diseases, Zealand University Hospital, Roskilde, Denmark; j Department of Infectious Diseases, Aalborg University Hospital, Aalborg, Denmark; k Department of Clinical Medicine, Aalborg University Hospital, Aalborg, Denmark; l Department of Clinical Medicine, Faculty of Health and Medical Sciences, University of Copenhagen, Denmark; m Department of Cardiology, Copenhagen University Hospital–Rigshospitalet, Copenhagen, Denmark; n Respiratory Medicine Section, Department of Medicine, Copenhagen University Hospital–Herlev and Gentofte, Copenhagen, Denmark; o Epidemiological Infectious Disease Preparedness, Statens Serum Institut, Copenhagen, Denmark; p Steno Diabetes Center Copenhagen, Copenhagen, Denmark

**Keywords:** behavioral science, diabetes, implementation, influenza, nudging, randomized controlled trial, registry, vaccination

## Abstract

**Background:**

Despite evidence demonstrating that influenza vaccination is associated with reduced risk of cardiovascular events and all-cause mortality in individuals with diabetes mellitus (DM), vaccine uptake remains suboptimal.

**Objectives:**

The purpose of this study was to assess the effectiveness of electronically delivered nudges on influenza vaccine uptake according to the presence of DM status versus other chronic diseases.

**Methods:**

NUDGE-FLU-CHRONIC was a nationwide, randomized, pragmatic implementation trial among younger and middle-aged (18-64 years) Danish citizens with chronic disease during the 2023/2024 influenza season. Participants were randomized in a 2.45:1:1:1:1:1:1 ratio to usual care (no electronic letter) or 1 of 6 different electronic nudge letters. The endpoint was receipt of a seasonal influenza vaccine on or before January 1, 2024.

**Results:**

Of 299,881 participants, 57,666 (19.2%) had DM (median age: 51.6 years, 43.0% female). During the season, 43.0% of those with DM vs 34.6% of those without DM received the vaccine (*P* < 0.001). Any electronic letter vs usual care was highly effective in increasing vaccine uptake in participants with DM (45.6% vs 36.5%, difference: +9.1 percentage points [99.29% CI: 7.9-10.3], relative risk ratio: 1.42 [99.29% CI: 1.39-1.44]). However, DM status modified the effect of the interventions such that participants without DM at baseline experienced a greater relative gain than those with DM (37.3% vs 25.9%, difference: +12.3 percentage points [99.29% CI: 11.7-12.8], risk ratio: 1.47 [99.29% CI: 1.45-1.50]; *P*_interaction_<0.001).

**Conclusions:**

Electronic nudge letters effectively boosted vaccine uptake in individuals with DM and in individuals free of DM but with other chronic diseases, but the effect was lower among those with DM. Electronic nudges represent a low-cost and effective strategy to boost influenza vaccination rates in the DM population. (Nationwide Utilization of Danish Government Electronic Letter System for Increasing InFLUenza Vaccine Uptake Among Adults With Chronic Disease; NCT06030739)

Individuals with diabetes mellitus (DM) have a greater susceptibility to seasonal influenza and face an increased risk of severe complications from influenza infection.^1^^,^^2^ In persons with DM, seasonal influenza vaccination has been shown to be associated with a reduced risk of mortality and hospitalization for severe diabetes-related complications including ketoacidosis, hypoglycemic episodes, and coma.^3^^,^^4^ Despite this evidence, and recommendations by public health authorities^5^ and patient organizations including the American Diabetes Association^6^, influenza vaccine uptake remains suboptimal in persons with DM. In Denmark, seasonal influenza uptake in persons with diabetes aged 65 years or below was only 40.7% in the 2022/2023 season^7^ and approximately 57% in the United States from 2007/2008 through 2017/2018 influenza seasons.^8^ In persons with DM, seasonal influenza vaccine uptake has been shown to be consistently lower in younger and middle-aged adults compared to older adults^7^^,^^8^—in Denmark the influenza vaccination rate among individuals with DM aged 65 or above was 82.9% in the 2022/2023 season.^7^ Effective and easily implementable strategies are therefore urgently needed to boost influenza vaccination uptake in this at-risk population.

Behavioral nudges in the form of letters or short messages have in previous studies demonstrated to be effective in boosting vaccination rates.^9^, ^10^, ^11^ In the NUDGE-FLU trial, which investigated the effect of electronic nudges on influenza vaccine uptake in adults ≥65 years during the 2022/2023 influenza season, we observed that DM status modified the effect of electronic nudges such that the benefits were greater in persons without DM.^7^ However, the baseline influenza vaccination rate in this population was >80% and the proportion receptive to nudging was therefore of limited size. The NUDGE-FLU-CHRONIC (Nationwide Utilization of Danish Government Electronic Letter System for Increasing InFLUenza Vaccine Uptake Among Adults With Chronic Disease) trial^12^ was specifically designed to investigate the effect of behavioral electronic nudge letters on influenza vaccine uptake in younger and middle-aged individuals (aged 18-64 years) with chronic diseases—a population eligible for influenza vaccination free-of-charge in Denmark.

In this prespecified analysis of the NUDGE-FLU-CHRONIC trial, we specifically aimed to assess the effectiveness of electronically delivered nudges on influenza vaccine uptake according to the presence of DM status versus other chronic diseases and to evaluate whether a boost to vaccine uptake would lead to a reduced risk of downstream cardiorespiratory clinical outcomes.

## Methods

### Study design and oversight

The design, rationale, and main results for the NUDGE-FLU-CHRONIC trial have previously been published.^12^^,^^13^ Briefly, it was a pragmatic, nationwide, registry-based implementation trial which randomized all eligible Danish citizens aged 18 to 64 years with a chronic disease qualifying for free-of-charge influenza vaccination through the national governmental vaccination program^14^ during the 2023/2024 influenza season in Denmark. The primary objective of the trial was to investigate the effect of electronic behavioral nudges on seasonal influenza vaccine uptake among patients with chronic diseases aged 18 to 64 years. The present study is a prespecified exploratory analysis of the NUDGE-FLU-CHRONIC trial focused on the effect of electronic behavioral nudges according to DM status. The Committees on Health Research Ethics in the Capital Region of Denmark (journal no. H-20080104) determined that the trial did not require ethical approval nor informed consent as the trial did not restrict the autonomy of the participants. The data authority in the Capital Region of Denmark approved the trial (approval no. P-2021 to 509) and the Danish National Health Data Authority approved and provided access to nationwide registry data (project ID: FSEID-00005848).

The first, second, and last author had unrestricted data access. All authors have critically reviewed and approved the manuscript. All authors made the decision to submit the manuscript and attest to the fidelity of the trial to the protocol.

#### Participants, blinding, and randomization

The Danish nationwide health registries were used to identify all Danish citizens aged ≥18 years to <65 years with a chronic disease known to be associated with an increased risk of severe complications from influenza infection and who therefore are eligible for influenza vaccination free-of-charge as part of the Danish governmental vaccination program.^14^ Trial inclusion is depicted in Supplemental Figure 1. The choice of registry-based disease-specific criteria was guided by recommendations from Statens Serum Institut, the national infectious disease preparedness and control governmental institution, and included chronic lung disease, chronic cardiovascular (CV) disease other than hypertension, type 1 DM (T1DM) or type 2 DM (T2DM), congenital or acquired immunodeficiency, impaired breathing due to muscular weakness, chronic renal or hepatic insufficiency, or other chronic conditions with an increased risk of severe influenza as determined by the treating physician. A full list of the registry-based criteria for the disease-specific definitions have previously been published.^13^

Participants were randomly allocated in a 2.45:1:1:1:1:1:1 ratio using simple unstratified randomization to either usual care or 1 of 6 intervention groups receiving different versions of behaviorally informed electronic letters nudging participants to get the seasonal influenza vaccination. The allocation ratio was based on the Dunnett allocation ratio to ensure maximal statistical power for between-group comparisons.^15^ Participants were not formally blinded to the intervention, but the usual care group was unaware that they constituted the control group in a randomized trial.

### Intervention

The different intervention letters were all based on the same standard letter template (Supplemental Figure 2) written in Danish with a highlighted variable section phrased according to behavioral science principles. All intervention letters included a link to book a vaccine appointment. There were 6 different active intervention arms: 1) standard letter; 2) standard letter sent at baseline and again after 10 days (repeated letter); 3) CV gain-framing; 4) respiratory disease gain-framing; 5) active choice/implementation prompt; 6) loss-framing letter (variable sections included in the different letters are listed in Supplemental Table 1). The rationale for the choice of letters were based on findings from the NUDGE-FLU trial.^16^ Intervention letters were delivered on September 24, 2023—a week before the governmental influenza vaccination began (the repeated letter was delivered on October 4, 2023). All letters included a link to the official vaccination booking website and a phone number which could also be used for booking. The intervention letters were delivered via the official governmental electronic letter system (Digital Post) used primarily by the Danish authorities, banks, and hospitals for communication with citizens. Danish citizens ≥15 years automatically receive official correspondence through Digital Post but can apply for exemption and instead receive the correspondence physically. Digital Post exemption criteria include physical or mental disability preventing use of the system, lack of access to computer/smartphone, limited command in Danish language, residence in an area without internet connection. Digital Post can be accessed through web browsers and a smartphone app with notifications on new correspondence sent to each citizen’s private e-mail and as text message. The sender of the intervention letter varied according to each participant’s region of residence (each sender was a named infectious disease attending physician from a regional university hospital). The usual care group did not receive any letter as part of the trial but were subject to any other public health campaign regarding influenza vaccination.

### Baseline data and endpoints

All data for the trial were retrieved through the nationwide Danish administrative health registries. Baseline and endpoint data were obtained using prespecified definitions based on International Classification of Diseases-10th edition (ICD-10) codes, Anatomical Therapeutical Chemical codes, and procedural codes (Supplemental Tables 2 and 3). In the present prespecified subgroup analysis, T1DM was defined based on ICD-10 code (E10) and was defined as either a relevant ICD-10 code (E11-E14) registered within 5 years prior to study baseline or use of glucose-lowering therapy (Anatomical Therapeutical Chemical code: A10 excluding A10BK (sodium-glucose co-transporter 2 inhibitors [SGLT2i]) and glucagon-like peptide-1 receptor agonists [GLP-1 RA]) unless the individual had also claimed prescription on another glucose-lowering drug (other than SGLT2i/GLP-1 RA) within 5 years prior to study baseline. This was done due to the use of SGLT2i, and GLP-1 RA for indications other than T2DM. DM duration was defined as the time from first DM diagnosis (ICD-10 codes: E10-E14), or first glucose-lowering prescription claim (whichever came first) and study baseline. The latest hemoglobin A1c (HbA1c) and estimated glomerular filtration rate measured within 6 months prior to study baseline were retrieved from the Registry of Laboratory Results for Research^17^ for all participants with DM.

In NUDGE-FLU-CHRONIC, the primary endpoint was receipt of an influenza vaccine on or before January 1, 2024, retrieved through the Danish Vaccination Registry.^18^

Prespecified exploratory clinical outcomes in NUDGE-FLU-CHRONIC included CV and respiratory hospitalizations, all-cause hospitalizations, all-cause death, and primary care utilization. All participants were followed for clinical outcomes from the date of initial intervention delivery (September 24, 2023) until May 31, 2024. The definitions for the clinical outcomes are listed in Supplemental Table 4.

### Statistical analysis

The statistical comparisons performed as part of this prespecified exploratory analysis adhered to the intention-to-treat-principle. As NUDGE-FLU-CHRONIC was not powered for this exploratory analysis, our results should be considered hypothesis-generating only. Baseline characteristics were presented as means with standard deviations or as proportions with percentage. The significance level was adjusted using the Bonferroni method where a total alpha of 0.05 was split evenly across all 7 coprimary comparisons resulting in an alpha level of 0.0071 and a corresponding 99.29% CI. CIs reported for comparisons of intervention arms in the study are the 99.29% CI. Statistical significance for all statistical tests were defined as a 2-tailed *P* value <0.0071. For the primary outcome, the absolute difference in proportions along with crude relative risk ratios (RRs) and adjusted CIs are presented according to DM status. Chi-square tests were used to derive unadjusted *P* values. Effect modification by DM status was assessed in a pooled analysis of all intervention arm vs usual care using binomial regression with identity link, covariates (randomization group [any letter vs no letter] and DM status), and an interaction term between randomization group and DM status. A similar approach was used when testing for effect modification by DM subgroups (T1DM or T2DM, insulin treatment, chronic kidney disease [CKD], chronic CV disease, DM duration [median], GLP-1 RA/SGLT2i treatment at baseline, and HbA1c level [median]). The individual DM subgroups were analyzed in respective models. Unadjusted *P* values derived from binomial regressions were reported for all tests of heterogeneity. Testing for effect modification by DM status was prespecified in the trial protocol.

For the exploratory assessment of clinical outcomes in participants with DM, comparisons were made in a pooled analysis of all intervention arm vs usual care. Time-to-event endpoints were assessed using Cox proportional hazards regression and participants were followed from date of initial intervention delivery until the first occurring of; end of follow-up (May 31, 2024), death, or emigration. The number of general primary care contacts was assessed using negative binomial regression. As the clinical outcomes were exploratory, only estimates with CIs are reported without formal hypothesis testing.

Statistical analyses were performed using SAS Software, version 9.4 (SAS Institute) and Stata MP, version 18.0 (StataCorp).

## Results

Of the 299,881 participants randomized in NUDGE-FLU-CHRONIC, 57,666 (19.2%) had DM at baseline (25,610 with T1DM [44.4%] and 32,056 with T2DM [55.6%]). The median age of participants with DM was 53.5 years (IQR: 42.1-59.6 years), 43.0% were female, median DM duration was 10.0 years (IQR: 4.4-15.2 years), and insulin was part of treatment in 51.1%. At baseline, GLP-RA or SGLT2i was part of treatment in 40.9% of participants and median HbA1c was 53 mmol/mol (IQR: 46-63 years). Baseline characteristics according to DM status are shown in Table 1. Briefly, participants with DM as compared to participants without, were older, more frequently male, and had a higher prevalence of CV comorbidities and risk factors (ischemic heart disease, cerebrovascular disease, peripheral vascular disease, hypertension, dyslipidemia, CKD, and antithrombotic medication). Also, participants with DM were more likely to have received a seasonal influenza vaccination in the previous season 2022/2023 northern hemisphere influenza season (40.9% vs 29.7%, *P* < 0.001). Baseline characteristics stratified according to DM status were well-balanced in the usual care group and any nudge letter group (Table 2). Participants with DM who were included were generally younger, had fewer comorbidities, and had a lower prevalence of T1DM than individuals with DM who were exempted from the Digital Post system and who could therefore not be included in the trial (Supplemental Table 5).

**Table 1:** Baseline Characteristics According to DM Status

	Participants With Diabetes (n = 57,666)	Participants Without Diabetes (n = 242,215)	*P* Value
Age, y	53.5 (42.1-59.6)	51.6 (39.4, 58.8)	<0.001
Female	24,784 (43.0%)	134,670 (55.6%)	<0.001
Influenza vaccination in previous season	23,566 (40.9%)	71,913 (29.7%)	<0.001
Chronic cardiovascular disease^a^	14,539 (25.2%)	70,199 (29.0%)	<0.001
Ischemic heart disease	7,027 (12.2%)	20,169 (8.3%)	<0.001
Heart failure	2,908 (5.0%)	8,819 (3.6%)	<0.001
Cerebrovascular disease	2,692 (4.7%)	8,375 (3.5%)	<0.001
Peripheral vascular disease	887 (1.5%)	1,259 (0.5%)	<0.001
Hypertension	32,643 (56.6%)	73,628 (30.4%)	<0.001
Dyslipidemia	30,060 (52.1%)	45,635 (18.8%)	<0.001
Chronic kidney disease	5,861 (10.2%)	9,347 (3.9%)	<0.001
Chronic lung disease	5,600 (9.7%)	44,739 (18.5%)	<0.001
Cancer	4,536 (7.9%)	47,990 (19.8%)	<0.001
Immunodeficiency	3,519 (6.1%)	25,758 (10.6%)	<0.001
HbA1c, mmol/mol^a^	53 (46-63)	36 (34-39)	<0.001
eGFR, mL/min/1/. 73 m^2^^b^	97.6 (83.0-107.4)	94.7 (81.4-104.7)	<0.001
Antithrombotic medication	13,628 (23.6%)	37,608 (15.5%)	<0.001
Type 1 diabetes mellitus	25,610 (44.4%)	NA	NA
Diabetes duration, y	10.0 (4.4, 15.2)	NA	NA
Insulin treatment	29,496 (51.1%)	NA	NA
GLP-1 RA or SGLT2i treatment	23,570 (40.9%)	NA	NA
Number of antidiabetic drugs			
No glucose-lowering medication	4,203 (7.3%)	NA	NA
1 glucose-lowering medications	31,417 (54.5%)	NA	NA
2 glucose-lowering medications	13,091 (22.7%)	NA	NA
≥3 glucose-lowering medications	8,955 (15.5%)	NA	NA

Values are median (IQR) or n (%).

DM = diabetes mellitus; eGFR = estimated glomerular filtration rate; GLP-1 RA = glucagon-like peptide 1 receptor agonist; HbA1c = hemoglobin A1c; SGLT2i = sodium-glucose co-transporter 2 inhibitors.

a HbA1c was available in 75,248 (31.1%) of participants without DM and in 43,135 (74.8%) of participants with DM.

b eGFR was available in 144,851 (59.8%) of participants without DM and in 41,080 (71.2%) of participants with DM.

**Table 2:** Baseline Characteristics According to Usual Care or Any Letter by DM Status

	Any Letter DM (n = 40,947)	Usual Care DM (n = 16,719)	Any Letter No DM (n = 171,875)	Usual Care No DM (n = 70,340)
Age, y	53.5 (42.1-59.6)	53.6 (42.1-59.7)	51.6 (39.4-58.8)	51.6 (39.4-58.8)
Female	17,619 (43.0%)	7,165 (42.9%)	76,477 (44.5%)	31,068 (44.2%)
Influenza vaccination in previous season	16,720 (40.8%)	6,846 (40.9%)	51,054 (29.7%)	20,859 (29.7%)
Chronic cardiovascular disease^a^	10,278 (25.1%)	4,261 (25.5%)	49,891 (29.0%)	20,308 (28.9%)
Ischemic heart disease	4,969 (12.1%)	2,058 (12.3%)	14,305 (8.3%)	5,864 (8.3%)
Heart failure	2,063 (5.0%)	845 (5.1%)	6,249 (3.6%)	2,570 (3.7%)
Cerebrovascular disease	1,897 (4.6%)	795 (4.8%)	5,971 (3.5%)	2,404 (3.4%)
Peripheral vascular disease	623 (1.5%)	264 (1.6%)	904 (0.5%)	355 (0.5%)
Hypertension	23,167 (56.6%)	9,476 (56.7%)	52,094 (30.3%)	21,534 (30.6%)
Dyslipidemia	21,335 (52.1%)	8,725 (52.2%)	32,469 (18.9%)	13,166 (18.7%)
Chronic kidney disease	4,205 (10.3%)	1,656 (9.9%)	6,660 (3.9%)	2,687 (3.8%)
Chronic lung disease	4,012 (9.8%)	1,588 (9.5%)	31,772 (18.5%)	12,967 (18.4%)
Cancer	3,197 (7.8%)	1,339 (8.0%)	34,086 (19.8%)	13,904 (19.8%)
Immunodeficiency	2,533 (6.2%)	986 (5.9%)	18,276 (10.6%)	7,482 (10.6%)
HbA1c, mmol/mol^a^	53 (46-63)	53 (46-63)	36 (34-39)	36 (34-39)
eGFR, mL/min/1/. 73 m^2^^b^	97.6 (83.0-107.5)	97.5 (83.0-107.3)	94.7 (81.4-104.7)	94.7 (81.2-104.7)
Antithrombotic medication	9,653 (23.6%)	3,975 (23.8%)	26,604 (15.5%)	11,004 (15.6%)
Type 1 diabetes mellitus	18,145 (44.3%)	7,465 (44.6%)	NA	NA
Diabetes duration, y	10.0 (4.4-15.2)	9.9 (4.4-15.2)	NA	NA
Insulin treatment	20,942 (51.1%)	8,554 (51.2%)	NA	NA
GLP-1 RA or SGLT2i treatment	16,746 (40.9%)	6,824 (40.8%)	NA	NA
Number of antidiabetic drugs				
No glucose-lowering medication	3,032 (7.4%)	1,171 (7.0%)	NA	NA
1 glucose-lowering medications	22,257 (54.4%)	9,160 (54.8%)	NA	NA
2 glucose-lowering medications	9,276 (22.7%)	3,815 (22.8%)	NA	NA
≥3 glucose-lowering medications	6,382 (15.6%)	2,573 (15.4%)	NA	NA

Values are median (IQR) or n (%).

Abbreviations as in Table 1.

a HbA1c was available in 75,248 (31.1%) of participants without DM and in 43,135 (74.8%) of participants with DM.

b eGFR was available in 144,851 (59.8%) of participants without DM and in 41,080 (71.2%) of participants with DM.

### Vaccination rates by DM status

**Figure 1:**
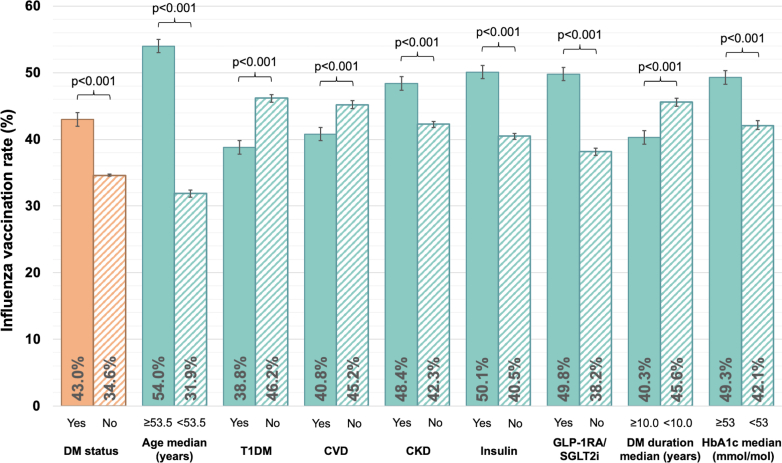
**Influenza Vaccination Rates Stratified According to DM Status and Subgroups of Individuals With DM** Bar diagram displaying influenza vaccination rates according to DM status (orange bars) and DM subgroups (turquoise bars) with 95% CIs. All subgroups are subgroups of individuals with DM only. *P* values for differences were computed using chi-square tests. CVD = chronic cardiovascular disease; CKD = chronic kidney disease; DM = diabetes mellitus; GLP-1 RA = glucagon-like peptide 1 receptor agonist; HbA1c = hemoglobin A1c; SGLT2i = sodium-glucose co-transporter-2 inhibitor; T1DM = type 1 diabetes mellitus.

Baseline characteristics were well-balanced across all 7 randomization arms in the participants with DM included in the trial (Supplemental Table 6). During follow-up, participants with DM more often received the seasonal influenza vaccination than participants without (43.0% [95% CI: 42.6-43.4] vs 34.6% [95% CI: 34.4-34.7], *P* < 0.001) (Figure 1). In participants with DM, seasonal influenza vaccine uptake was higher in older participants and those with CKD, insulin use, GLP-1 RA/SGLT2i use, and higher HbA1c level, and lower in those with T1DM, chronic CV disease, and shorter DM duration (Figure 1).

### Effects of interventions

**Figure 2:**
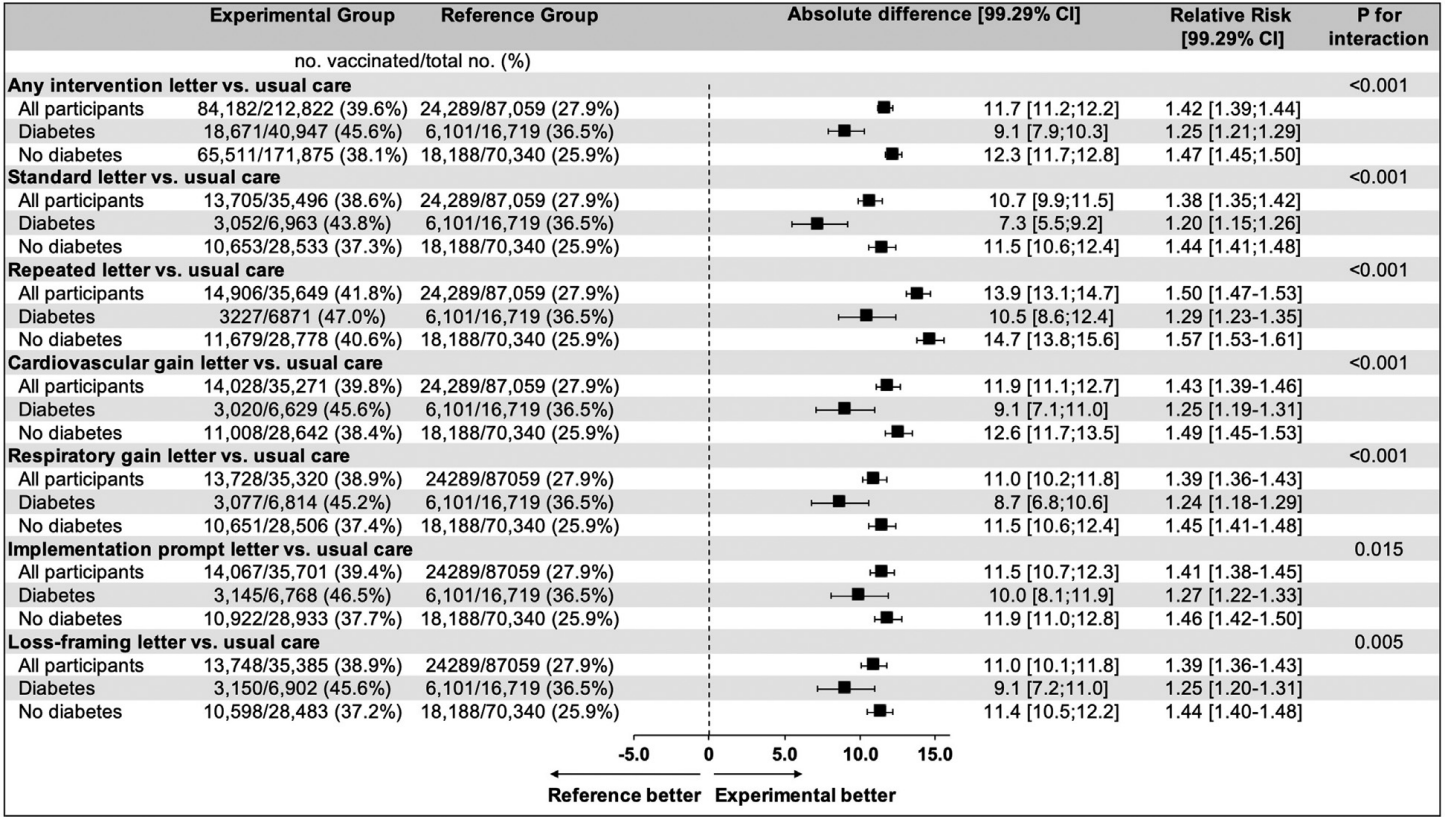
**Effects of Electronic Letters on the Receipt of an Influenza Vaccine Stratified According to DM Status** A total of 7 comparisons were made with the last comparison being any electronic nudge letter compared to usual care. Comparisons were stratified according to DM status at baseline. Alpha-adjusted (99.29%) CIs are provided. Statistical significance was defined a *P* value <0.0071. The *P* values for interaction were computed using binomial regression with identity link and an interaction term. Electronic letters in each active intervention were based on a standard letter with a variable component.

In participants with DM at baseline, any electronic letter (n = 40,947) vs usual care (n = 16,719) was highly effective in increasing vaccine uptake (45.6% vs 36.5%, difference: +9.1 percentage points [99.29% CI: 7.9-10.3], relative RR: 1.25, 99.29% CI: [1.21-1.29]), corresponding to a number needed to nudge of 11. However, DM status modified the effect of any electronic letter such that participants without DM at baseline experienced a greater relative gain than those with DM (38.1% vs 25.9%, difference: +12.3 percentage points [99.29% CI: 11.7-12.8], RR: 1.47 [99.29% CI: 1.45-1.50]; *P*_interaction_ <0.001) (Figure 2).

Overall, each of the individual types of nudge letters tested was effective in boosting vaccine uptake in both participants with and without DM at baseline (Central Illustration). As with the pooled analysis of any letter vs usual care, a significant heterogeneity with DM status was observed for all types of electronic letters tested in the trial such that the interventions in all cases resulted in slightly lower relative gains in participants with DM (all *P*_interaction_ <0.015) (Figure 2). In participants with DM at baseline, the numerically largest absolute effects were observed with the repeated letter (47.0% vs 36.5%; difference: +10.5 percentage points [99.29% CI: 8.6-12.4], RR: 1.29 [99.29% CI: 1.23-1.35]), the implementation prompt letter (46.5% vs 36.5%; difference: +10.0 percentage points [99.29% CI: 8.1-11.9], RR: 1.27 [99.29% CI: 1.22-1.33]), and the CV gain letter (45.6% vs 36.5%; difference: +9.1 percentage points [99.29% CI: 7.1-11.0], RR: 1.25 [99.29% CI: 1.19-1.31]) whereas the most effective nudge letters in participants without DM at baseline were the repeated letter and the CV gain letter (Figure 2).

**Figure 3:**
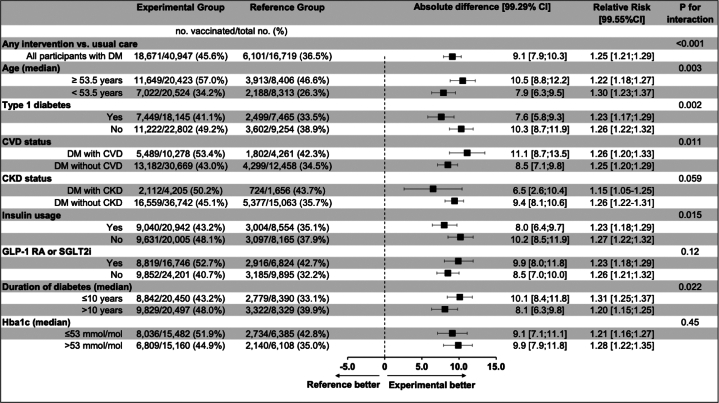
**Effect of Any Electronic Nudge Letter Versus Usual Care Across DM Subgroups** DM subgroup analyses were performed with all 6 intervention arms pooled (any electronic letter) versus usual care. Comparisons were stratified according to DM status at baseline. Alpha-adjusted (99.29%) CIs are provided. statistical significance was defined a *P* Value <0.0071. The *P* values for interaction were computed using binomial regression with identity link and an interaction term. Abbreviations as in Figure 1.

While the directionality of effect favored any electronic letter vs usual care in all DM subgroups, significant heterogeneity was observed for several DM subgroups (Figure 3). Incremental relative effectiveness was suggested among older participants with DM (≥median of 53.5 years) (*P*_interaction_ = 0.003), and those who had T2DM (as compared to T1DM) (*P*_interaction_ = 0.002) (Figure 3).

**Central Illustration:**
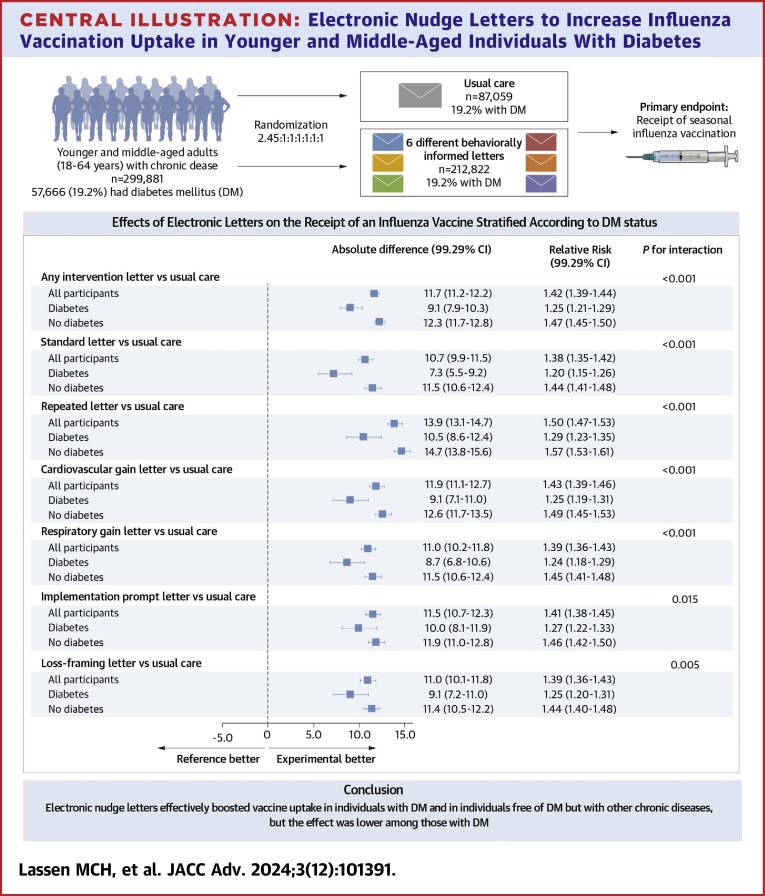
**Electronic Nudge Letters to Increase Influenza Vaccination Uptake in Younger and Middle-Aged Individuals With Diabetes** A total of 7 comparisons were made with the last comparison being any electronic nudge letter compared to usual care. Comparisons were stratified according to DM status at baseline. Alpha-adjusted (99.29%) CIs are provided. statistical significance was defined a *P* value <0.0071. The *P* values for Interaction were computed using binomial regression with identity link and an interaction term. Electronic letters in each active intervention were based on a standard letter with a variable component.

### Clinical outcomes

Results of the exploratory assessment of effects of any interventional letter vs usual care on risk of clinical outcomes among participants with DM are displayed in Table 3. Overall, the boost in seasonal influenza vaccination did not translate into substantial differences in clinical outcomes among participants with DM. Hospitalization for pneumonia or influenza occurred in 119 (0.7%) participants with DM in the usual care arm vs 303 (0.7%) of those receiving any interventional letter (HR: 1.04 [95% CI: 0.84-1.29]). Cardiorespiratory hospitalization occurred in 427 (2.6%) participants with DM in the usual care arm vs 1,001 (2.4%) of those receiving any interventional letter (HR: 0.95 [95% CI: 0.41-1.07]). Death from any cause occurred in 147 (0.9%) participants with DM in the usual care arm vs 367 (0.9%) of those receiving any interventional letter (HR: 1.02 [95% CI: 0.84-1.24]).

**Table 3:** Clinical Outcomes for Comparison of Any Intervention Letter Vs Usual Care Among Participants With DM

	Usual Care DM (n = 16,719)	Any Intervention Letter DM (n = 40,947)	Hazard Ratio or Rate Ratio (95% CI)
Hospitalization for pneumonia or influenza	119 (0.7)	303 (0.7)	1.04 (0.84-1.29)
Respiratory hospitalization	220 (1.3)	493 (1.2)	0.91 (0.78-1.07)
Cardiovascular hospitalization	218 (1.3)	537 (1.3)	1.01 (0.86-1.18)
Cardiorespiratory hospitalization	427 (2.6)	1,001 (2.4)	0.95 (0.41-1.07)
Incident HF, HF hospitalization, or CV death	109 (0.7)	214 (0.5)	0.80 (0.64-1.01)
Myocardial infarction, stroke, coronary revascularization, or CV death	147 (0.9)	355 (0.9)	0.98 (0.81-1.19)
All-cause hospitalization	2,224 (13.3)	5,611 (13.7)	1.03 (0.98-1.08)
All-cause mortality	147 (0.9)	367 (0.9)	1.02 (0.84-1.24)
Number of general practitioner contacts	1 (0-4)	1 (0-4)	0.99 (0.96-1.02)

Values are no. of events (%) or median (IQR). The prespecified registry-based endpoint definitions are listed in Supplemental Table 4. Effect estimates are reported as hazard ratios for all outcomes besides the number of general practitioner contacts, for which the effect is reported as a rate ratio. Hazard ratios were generated from Cox proportional hazards models, whereas the rate ratio was calculated using negative binomial regression. Vaccination visits were excluded from the number of general practitioner contacts.

CV = cardiovascular; HF = heart failure; other abbreviation as in Table 1.

## Discussion

Seasonal influenza vaccination rates in younger and middle-aged individuals with DM remain suboptimal in Denmark with an overall vaccination rate of just 43.0% despite the vaccine being offered free-of-charge as part of the governmental vaccination program. In this prespecified analysis of the NUDGE-FLU-CHRONIC trial, any electronic nudge letter, as compared with usual care, was highly effective in boosting influenza vaccination rates in participants with DM with absolute effects sizes ranging between 7 and 11 percentage points and a number needed to nudge of 11 for any interventional letter. However, DM status modified the effect such that participants without DM experienced an even greater effect than those with DM. In participants with DM, all individual letter types effectively increased influenza vaccination rates but the 2 most effective nudge letters were the repeated letter and the implementation prompt letter. The nudge letters remained effective across DM subgroups.

In the present trial, any electronic nudge letter was found to be highly effective in boosting influenza vaccination rates in participants with DM. Compared with the usual care vaccination rate, the combined 6 intervention arms resulted in an additional 3,700 (9.1 percentage points) vaccinated individuals with DM corresponding to a number needed to nudge of 11 to boost seasonal vaccination uptake by 1. The electronic nudge letters represent a low-cost and easily scalable implementation strategy which may improve health behavior in individuals with DM. Use of the most effective letters on a nationwide level would have resulted in an even larger number of additional vaccines with DM.

We observed that electronic nudge letters were more effective in increasing influenza vaccination rates in participants with DM than what was previously observed in the similar NUDGE-FLU trial conducted during the 2022/2023 influenza season in Danish citizens aged ≥65 years.^7^ Various factors may help explain the large differences observed between the 2 trials. The difference in the usual care influenza vaccination rates differed substantially as it was 83.0% among participants with DM in NUDGE-FLU compared to only 43.0% in NUDGE-FLU-CHRONIC, and thus the proportion of participants receptive to nudging was much larger in the present trial. This confirms prior theory suggesting a larger effect of nudging interventions when there is a low uptake of the desired health behavior at baseline in the studied population. In NUDGE-FLU, the usual care group all received an informational letter outside of the trial informing them on influenza vaccine eligibility from the Danish Health Authority due to them being ≥65 years. Such a letter is not sent to citizens <65 years despite eligibility for influenza vaccination free-of-charge. Of course, the population in the present trial was still subject to other governmental influenza vaccination campaigns, but direct messaging has previously been found to be the most effective strategy in relation to changing health-related behavior.^19^ It is possible that a large proportion of the present trial population may not have been aware that they were eligible for free-of-charge influenza vaccination, which may have greatly increased their receptiveness to nudging. Other reasons for the suboptimal vaccine uptake in younger and middle-aged individuals with DM possibly includes inadequate knowledge of the clinical benefits among both patients and health care providers, perception of low vulnerability to influenza, and other reasons for vaccine hesitancy.^20^^,^^21^ Therefore, the distinct populations enrolled in NUDGE-FLU and NUDGE-FLU-CHRONIC might have been influential on the differences in effect sizes observed for similar interventions.

In this prespecified analysis, we observed that any electronic nudge letter was less effective in participants with DM compared to those without. Such an observation was also made in the previous NUDGE-FLU trial, where the most effective electronic nudge letters (the repeated letter and the CV gain letter) significantly increased influenza vaccination rates by approximately 1 percentage point in participants without DM while the effect was smaller or even in the opposite direction, in those with DM.^7^^,^^16^ It is interesting that in both trials with total >1.2 million Danish citizens of which 180,000 had DM, DM status consistently modified the effect of electronic nudges such that the interventions were less effective in participants with DM. An important factor was that those with DM in the present study had a higher baseline vaccination rate than those without DM (43.0% vs 34.6%), and this apparent difference in the “nudge-able” proportions—which was also observed in NUDGE-FLU^7^—could be part of the explanation for why the interventions were generally less effective in those with DM. Other factors to consider is that individuals with DM constitute a very distinct group in the study population as a diagnosis of DM—especially a diagnosis of T1DM—entails long-term dramatic lifestyle changes including frequent routine health examinations at their general practitioner and diabetes outpatient clinic, diet and physical activity changes, blood glucose monitoring, chronic medications, foot care, and eye check-ups. During these visits, individuals with DM are frequently prompted to change health behavior and warned about the possible complications to their chronic disease. This may result in “nudging-fatigue” or possibly even psychological reactance where the individual chooses to act against given advice because they feel that their autonomy is threatened.^22^ This may also explain why we observed the electronic nudges to be even less effective in T1DM than in T2DM, as T1DM is generally diagnosed much earlier in life than T2DM and entails even more lifestyle changes than T2DM.

Even though DM status modified the effectiveness of the electronic nudges, it is important to highlight that the electronic nudges were still highly effective in boosting influenza vaccination rates, also in those with DM. Our results from the present trial together with the findings from NUDGE-FLU and previous nudging studies^22^, ^23^, ^24^ focused specifically on changing health behavior in individuals with DM underscores the difficulty in designing nudges to change health-related behavior in individuals with DM. Electronic nudges specifically tailored to individuals with DM to change health-related behavior should be explored in future trials.

While we observed that the interventional letters led to increases in seasonal influenza vaccine uptake in participants with DM, this did not seem to translate into any differences in risk of downstream cardiorespiratory clinical outcomes. For participants with DM, the largest nominal differences were observed for the heart failure-related outcome which favored the interventional letters. Possible reasons for why we did not observe effects on clinical outcomes may include: 1) our study population consisted of younger and middle-aged adults with relatively low event rates which limited statistical power for this analysis, and 2) the number of preventable events by influenza vaccination was possibly low as influenza infection may only have caused a minor number of excess clinical events.

### Study limitations

The present trial is subject to limitations. Firstly, the trial was conducted exclusively in Denmark using a mandatory governmental electronic mail system and the results may therefore not be generalizable to all parts of the world. However, it is likely that some common human psychological features would make the strategies effective in other countries, but it may only be feasible in countries in which a large proportion of the target population can be reached digitally. Secondly, the results of the present study only applies to younger and middle-aged adults with DM or other types of chronic disease, and we have previously published results on older adults (≥65 years) with DM from the NUDGE-FLU trial which suggests that the effectiveness of electronic nudges to improve influenza vaccination rates differ substantially between age-groups.^7^ However, the effect modification by DM status was consistent across both trials which highlights the need for nudges tailored specifically for individuals with DM. Lastly, baseline and outcome data were obtained exclusively through the national health registries which may be prone to misclassification, however, any such misclassification would be expected to be equally distributed across the intervention arms due to randomization.

## Conclusions

Electronic nudge letters significantly boosted vaccine uptake in a broad range of individuals with DM and in individuals free of DM but with other chronic diseases, but the effect was slightly lower among those with DM compared to those without. Even so, the electronic nudges represent a low-cost and scalable strategy to improve health care in participants with DM. Studies are needed to better understand nudging effects in individuals with DM and to assess the effect of electronic nudges tailored specifically to those with DM.

Perspectives **COMPETENCY IN MEDICAL KNOWLEDGE 1:** Electronic nudge letters compared with usual care effectively boost seasonal influenza vaccine uptake in individuals with DM.

**COMPETENCY IN MEDICAL KNOWLEDGE 2:** The beneficial effects of electronic nudge letters on seasonal influenza vaccine uptake did not significantly translate into differences in risk of downstream cardiorespiratory clinical outcomes.

**TRANSLATIONAL OUTLOOK 1:** The younger and middle-aged individual with diabetes can effectively be nudged to change health-related behavior with electronically delivered nudge letters.

**TRANSLATIONAL OUTLOOK 2:** The most effective electronic nudge letter strategies in individuals with diabetes are implementation prompts, repeated letters, and letters focused on CV gain framing.

**TRANSLATIONAL OUTLOOK 3:** Electronic nudge letters were more effective in individuals without diabetes than individuals with diabetes. Studies are needed to better understand nudging effects in individuals with diabetes and to assess the effect of electronic nudges tailored specifically to those with diabetes.

## Funding support and author disclosures

No funding was obtained for the NUDGE-FLU-CHRONIC trial. Dr Højbjerg Lassen was funded by a research grant from the Danish Heart Foundation (Grant no.: 21-R149-A10082-22194). Dr Biering-Sørensen has received research grants from 10.13039/100014588Sanofi Pasteur, 10.13039/100004330GSK, 10.13039/501100004191Novo Nordisk, 10.13039/100004325AstraZeneca, 10.13039/100008497Boston Scientific, and 10.13039/100006775GE Healthcare; consulting fees from Novo Nordisk, IQVIA, Parexel, Amgen, CSL Seqirus, GSK, and Sanofi Pasteur; and lecture fees from Bayer, Novartis, Sanofi Pasteur, GE healthcare, and GSK. Dr Vaduganathan has received research grant support or served on advisory boards for American Regent, Amgen, AstraZeneca, Bayer AG, Baxter Healthcare, Boehringer Ingelheim, Chiesi, Cytokinetics, Lexicon Pharmaceuticals, Novartis, Novo Nordisk, Pharmacosmos, Relypsa, Roche Diagnostics, Sanofi, and Tricog Health; has speaker engagements with AstraZeneca, Boehringer Ingelheim, Novartis, and Roche Diagnostics; and participates on clinical trial committees for studies sponsored by 10.13039/100004325AstraZeneca, 10.13039/100004326Bayer AG, Galmed, Occlutech, 10.13039/100004336Novartis, and 10.13039/100019443Impulse Dynamics. Dr Bhatt has received fees from Sanofi. Dr Claggett has received consulting fees from Amgen, Cardurion, Corvia, Myokardia, and Novartis. Dr Køber has received speaker fees from Novo Nordisk, Novartis, AstraZeneca, Boehringer Ingelheim, and Bayer. Dr Solomon has received research grants from Actelion, 10.13039/100006400Alnylam, 10.13039/100002429Amgen, 10.13039/100004325AstraZeneca, Bellerophon, 10.13039/100004326Bayer, 10.13039/100002491BMS, 10.13039/100008599Celladon, 10.13039/100014941Cytokinetics, Eidos, 10.13039/100005564Gilead, 10.13039/100004330GSK, Ionis, 10.13039/100004312Lilly, Mesoblast, 10.13039/100016619MyoKardia, 10.13039/100000002NIH/10.13039/100000050NHLBI, Neurotronik, 10.13039/100004336Novartis, 10.13039/501100004191Novo Nordisk, 10.13039/100019040Respicardia, 10.13039/100004339Sanofi, Theracos, US2.AI and consulted for Abbott, Action, Akros, Alnylam, Amgen, Arena, AstraZeneca, Bayer, Boehringer Ingelheim, BMS, Cardior, Cardurion, Corvia, Cytokinetics, Daiichi-Sankyo, GSK, Lilly, Merck, Myokardia, Novartis, Roche, Theracos, Quantum Genomics, Cardurion, Janssen, Cardiac Dimensions, Tenaya, Sanofi, Dinaqor, Tremeau, CellProThera, Moderna, American Regent, Sarepta, Lexicon, Anacardio, Akros, and Puretech Health. All other authors have reported that they have no relationships relevant to the contents of this paper to disclose.
