# Supplementary material for: Electronic Nudge Letters to Increase Influenza Vaccination Uptake in Younger and Middle-Aged Individuals With Diabetes
Source: JACC Adv. 2024 Nov 13;3(12):101391. doi: 10.1016/j.jacadv.2024.101391 (PMC11600668; doi:10.1016/j.jacadv.2024.101391)
Supplement: Supplemental data [file mmc1.docx]

**Supplemental Figure S1:** Study inclusion flowchart

**Caption:** Flowchart depicting identification, exclusion, and randomization of participants in NUDGE-FLU-CHRONIC


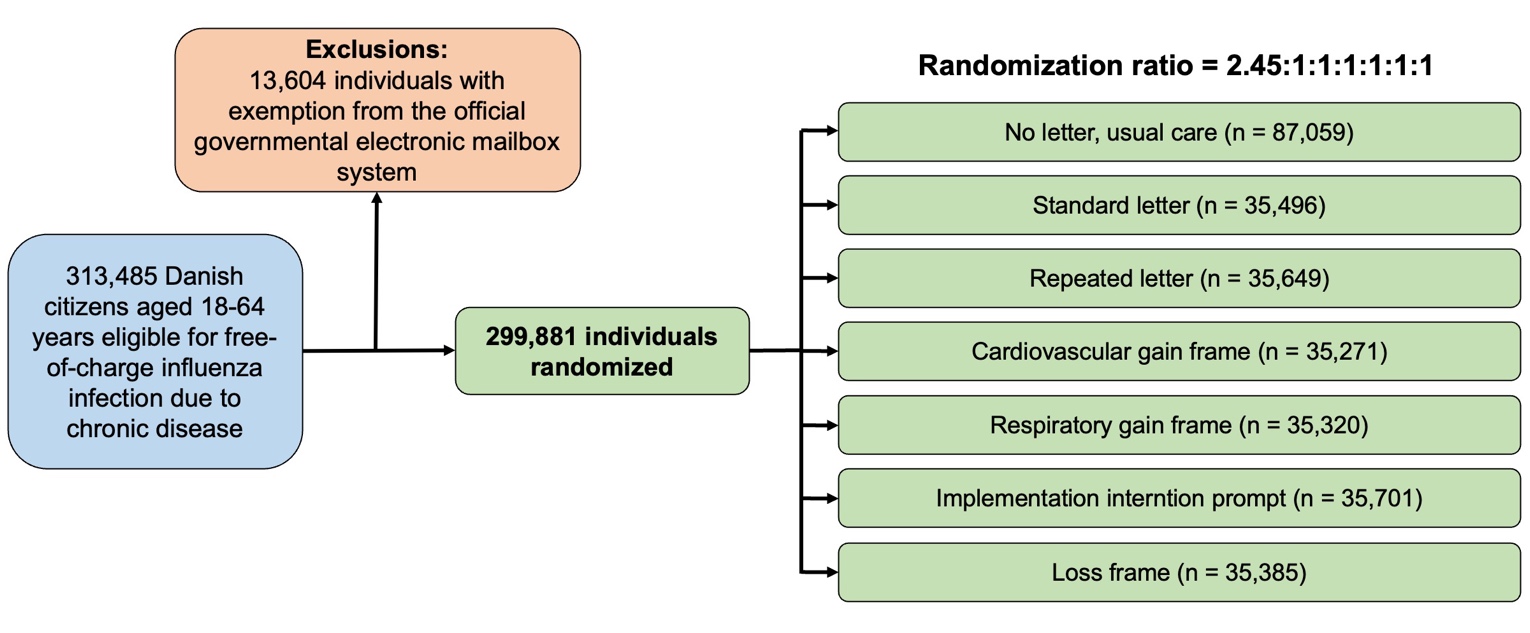


**Supplemental Figure S2:** Standard Letter

**Caption:** English translation of the standard letter template. Text-based nudges used in several intervention arms were added to the highlighted placeholder. Supplemental Table S1 displays an overview of the intervention arms including English translation of the specific nudges. All letters were written in Danish language.

**
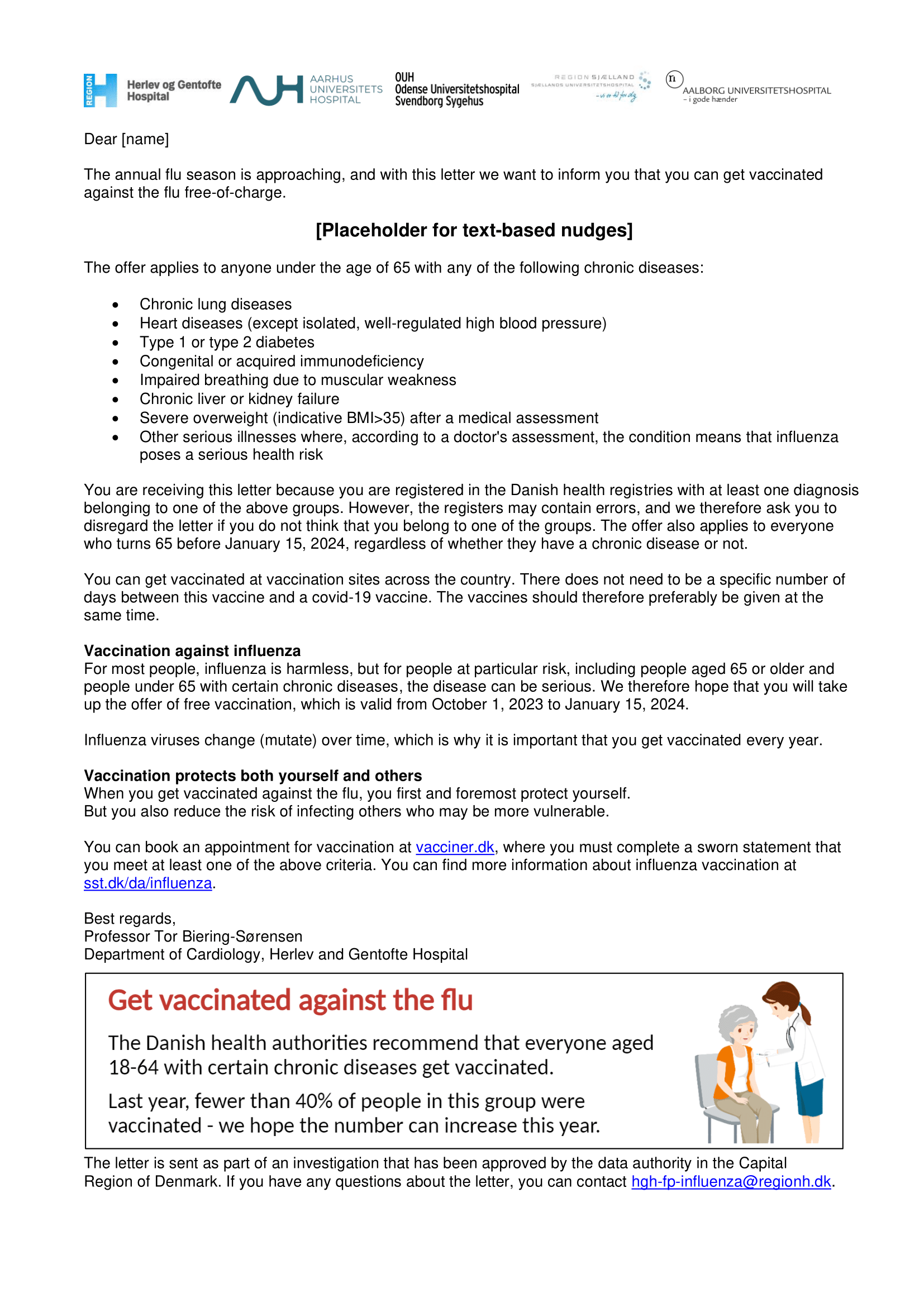
**

**Supplemental Table S1:** Overview of intervention arms

| **Study Arm** | **Description** | **Behavioral Science Concept** | **Text Added to Placeholder (English Translation)** |
| --- | --- | --- | --- |
| Usual care | The usual care group received no letter on influenza vaccination but were subject to other standard public health vaccination campaigns. | - | - |
| Standard letter | Standard informational letter (displayed in Supplemental Figure 1) | - | - |
| Repeated letter | Standard letter sent at baseline and repeated after 10 days | Priming and hot state activation | - |
| CV gain | Text added to standard letter | Gain-framing (cardiovascular) | *“In addition to its protection against influenza infection, influenza vaccination also seems to protect against cardiovascular disease such as heart attacks and heart failure.”* |
| Respiratory gain | Text added to standard letter | Gain-framing (respiratory) | *“In addition to its protection against influenza infection, influenza vaccination also seems to protect against severe pneumonia and worsening of chronic lung disease.”* |
| Implementation prompt | Text added to standard letter including highlighted booking link | Active choice/implementation intention prompt | *“We encourage you to record your appointment time here: _________. You can book your appointment at this link:* [*www.vacciner.dk*](http://www.vacciner.dk)*”* |
| Loss-framing | Text added to standard letter | Loss-framing | *“If you do not get vaccinated, your risk of getting influenza is increased, and you are also more likely to spread the disease to your loved ones.”* |

**Supplemental Table S2:** Pre-specified definitions of baseline conditions

| **Condition** | **ICD-10/ATC codes** | **Inpatient/outpatient/minimum required length of stay/other criteria** | **Timeframe** |
| --- | --- | --- | --- |
| Chronic lung disease | A15-A16, D860, E84, J42-J47, J84, Z942 | Any | ≤10 years prior to intervention delivery |
| Diabetes (ICD-10) | E10-E14 | Any | ≤10 years prior to intervention delivery |
| Diabetes (ATC) | A10 | ≥1 claimed prescription  IF only A10BJ or A10BK has been claimed ≤180 days prior to intervention delivery, then has to have claimed ≥1 prescription for another A10 drug ≤5 years | ≤180 days prior to intervention delivery |
| Hypertension (ICD-10) | I10-I15 | Any | ≤10 years prior to intervention delivery |
| Hypertension (ATC) | Diuretics: C03  b-blockers: C07  Calcium blockers: C08  Renin-angiotensin system inhibitors: C09 | ≥1 claimed prescription from ≥2 drug classes | ≤180 days prior to intervention delivery |
| Dyslipidemia (ATC) | C10 | ≥1 claimed prescription | ≤180 days prior to intervention delivery |
| Ischemic heart disease | I20-I25 | Any | ≤10 years prior to intervention delivery |
| Heart failure | I50 | Any | ≤10 years prior to intervention delivery |
| Cerebrovascular disease | I60-I69 | Any | ≤10 years prior to intervention delivery |
| Peripheral vascular disease | I70, I74 | Any | ≤10 years prior to intervention delivery |
| Chronic cardiovascular disease | I20-I28, I34-I37, I42-I50, I60-I69, I70, I74, Q20-Q26 | Any | ≤10 years prior to intervention delivery |
| Cancer | C00-C97 (not C44) | Any | ≤10 years prior to intervention delivery |
| Chronic kidney disease | E102, E112, E132, E142, I120, N02-N08, N11-N12, N14, N18-N19, N26, N158-N160, N162-N164, N168, M300, M313, M319, M321B, Q612-Q613, Q615, Q619, T858-T859, Z992 | Any | ≤10 years prior to intervention delivery |
| Immunodeficiency (ICD-10) | B20-B24, D80-D84, D89, O987, Z21, Z940-Z944, Z948A | Any | ≤10 years prior to intervention delivery |
| Immunodeficiency (ATC) | H02AB, L04 | ≥1 claimed prescription | ≤180 days prior to intervention delivery |

**Supplemental Table S3:** Pre-specified definitions for medication use at baseline

| **Medication** | **ATC codes** | **Number of prescriptions** | **Timeframe** |
| --- | --- | --- | --- |
| Antithrombotics | B01 | ≥1 claimed prescription | ≤180 days prior to intervention delivery |
| Insulin | A10A | ≥1 claimed prescription | ≤180 days prior to intervention delivery |
| Biduanide | A10BA | ≥1 claimed prescription | ≤180 days prior to intervention delivery |
| Sodium-glucose cotransporter 2 inhibitors | A10BK | ≥1 claimed prescription | ≤180 days prior to intervention delivery |
| Glucagon-like peptide 1 receptor agonists | A10BJ | ≥1 claimed prescription | ≤180 days prior to intervention delivery |
| Dipeptidyl peptidase-4 inhibitor | A10BH | ≥1 claimed prescription | ≤180 days prior to intervention delivery |
| Sulfonylurea | A10BB | ≥1 claimed prescription | ≤180 days prior to intervention delivery |

**Supplementary Table S4:** Prespecified definitions of exploratory clinical endpoints

| **Endpoint** | **ICD10/ATC/**  **procedural codes** | **Diagnosis type*** | **Inpatient/outpatient/minimum required length of stay/other criteria** | **Timeframe** |
| --- | --- | --- | --- | --- |
| Influenza or pneumonia hospitalization | J09-J18 | A | Inpatient, at least 1 night | From intervention delivery until May 31, 2024 |
| Respiratory hospitalization | J00-J06, J09-J18, J20-J22, J40-J47, J80-J81, J85-J86, J96 | A | Inpatient, at least 1 night | From intervention delivery until May 31, 2024 |
| Cardio-respiratory hospitalization | I11, I13, I20-I25, I30-I31, I33, I38-I42, I46-I50, I60-I69, I74, J00-J06, J09-J18, J20-J22, J40-J47, J80-J81, J85-J86, J96 | A | Inpatient, at least 1 night | From intervention delivery until May 31, 2024 |
| Cardiovascular hospitalization | I11, I13, I20-I25, I30-I31, I33, I38-I42, I46-I50, I60-I69, I74 | A | Inpatient, at least 1 night | From intervention delivery until May 31, 2024 |
| Any hospitalization | Any | Any | Inpatient, at least 1 night | From intervention delivery until May 31, 2024 |
| All-cause mortality | - | - | - | From intervention delivery until May 31, 2024 |
| Composite of incident heart failure, heart failure hospitalization, and cardiovascular death | Incident heart failure and heart failure hospitalization: I50  Deaths: Any I-diagnosis as cause of death | Incident heart failure and heart failure hospitalization: A | Heart failure hospitalization: Inpatient, at least 1 night  Incident heart failure: Any (only participants without known heart failure) | From intervention delivery until May 31, 2024 |
| Composite of myocardial infarction, revascularization, stroke, and cardiovascular death | Myocardial infarction and stroke: I21, I63-I64  Revascularization: KFNA, KFNB, KFNC, KFND, KFNE, KFNF, KFNG, KFNH, KFNJ, KFNK, KFNW  Deaths: Any I-diagnosis as cause of death | Myocardial infarction and stroke: A | Myocardial infarction and stroke: Inpatient, at least 1 night | From intervention delivery until May 31, 2024 |
| Number of contacts to general practitioner (excluding vaccination visit) | - | - | - | From intervention delivery until May 31, 2024 |

* A = primary discharge diagnosis; B = secondary discharge diagnosis. Endpoints are assessed using ICD-10 and procedural codes. ICD, International Classification of Diseases

**Supplemental Table S5:** Baseline Characteristics of trial population with diabetes included in the trial compared with those with diabetes who were excluded due to exemption from governmental electronic letter system

|  | **Excluded** | **Included** |
| --- | --- | --- |
| N | 4,439 | 57,666 |
| Age, median years (IQR) | 58.5 (53.1, 61.8) | 53.5 (42.1, 59.6) |
| Female sex, n (%) | 1,928 (43.4%) | 24,784 (43.0%) |
| Influenza vaccination in previous season, n (%) | 2,081 (46.9%) | 23,566 (40.9%) |
| Chronic cardiovascular disease*, n (%) | 1,688 (38.0%) | 14,539 (25.2%) |
| Ischemic heart disease, n (%) | 765 (17.2%) | 7,027 (12.2%) |
| Heart failure, n (%) | 389 (8.8%) | 2,908 (5.0%) |
| Cerebrovascular disease, n (%) | 491 (11.1%) | 2,692 (4.7%) |
| Hypertension, n (%) | 3,180 (71.6%) | 32,643 (56.6%) |
| Chronic kidney disease, n (%) | 683 (15.4%) | 5,861 (10.2%) |
| Chronic lung disease, n (%) | 779 (17.5%) | 5,600 (9.7%) |
| Cancer, n (%) | 372 (8.4%) | 4,536 (7.9%) |
| Immunodeficiency, n (%) | 357 (8.0%) | 3,519 (6.1%) |
| HbA1c, mmol/mol (IQR)* | 55 (46, 68) | 53 (46, 63) |
| eGFR, mL/min/1/. 73m^2^ (IQR)** | 96.1 (77.3, 104.9) | 97.6 (83.0, 107.4) |
| Type 1 diabetes mellitus, n (%) | 1,293 (29.1%) | 25,610 (44.4%) |
| Diabetes duration, median years (IQR) | 10.4 (4.9, 14.7) | 10.0 (4.4, 15.2) |
| Insulin treatment, n (%) | 1,793 (40.4%) | 29,496 (51.1%) |
| GLP-1 RA or SGLT2i treatment, n (%) | 2,009 (45.3%) | 23,570 (40.9%) |
| Number of antidiabetic drugs: |  |  |
| No glucose-lowering medication, n (%) | 462 (10.4%) | 4,203 (7.3%) |
| 1 glucose-lowering medications, n (%) | 1,890 (42.6%) | 31,417 (54.5%) |
| 2 glucose-lowering medications, n (%) | 1,149 (25.9%) | 13,091 (22.7%) |
| ≥3 glucose-lowering medications, n (%) | 938 (21.1%) | 8,955 (15.5%) |

**Supplemental Table S6:** Baseline characteristics according to randomization arm in participants with diabetes

|  | **Usual care** | **Standard letter** | **Repeated letter** | **CV gain** | **Respiratory gain** | **Implementation prompt** | **Loss-framing** |
| --- | --- | --- | --- | --- | --- | --- | --- |
| N | 16,719 | 6,963 | 6,871 | 6,629 | 6,814 | 6,768 | 6,902 |
| Age, median years (IQR) | 53.6 (42.1, 59.7) | 53.7 (42.3, 59.6) | 53.3 (41.6, 59.5) | 53.4 (42.3, 59.6) | 53.4 (41.8, 59.6) | 53.4 (42.6, 59.5) | 53.4 (41.8, 59.8) |
| Female sex, n (%) | 7,165 (42.9%) | 2,938 (42.2%) | 2,954 (43.0%) | 2,924 (44.1%) | 2,947 (43.3%) | 2,893 (42.8%) | 2,963 (42.9%) |
| Influenza vaccination in previous season, n (%) | 6,846 (41.0%) | 2,803 (40.3%) | 2,797 (40.7%) | 2,655 (40.1%) | 2,776 (40.7%) | 2,890 (42.7%) | 2,799 (40.6%) |
| Chronic cardiovascular disease*, n (%) | 4,261 (25.5) | 1,780 (25.6) | 1,684 (24.5) | 1,639 (24.7%) | 1,718 (25.2%) | 1,732 (25.6%) | 1,725 (25.0%) |
| Ischemic heart disease, n (%) | 2,058 (12.3%) | 882 (12.7%) | 797 (11.6%) | 777 (11.7%) | 819 (12.0%) | 845 (12.5%) | 849 (12.3%) |
| Heart failure, n (%) | 845 (5.1%) | 373 (5.4%) | 337 (4.9%) | 311 (4.7%) | 344 (5.1%) | 365 (5.4%) | 333 (4.8%) |
| Cerebrovascular disease, n (%) | 795 (4.8%) | 306 (4.4%) | 325 (4.7%) | 282 (4.3%) | 342 (5.0%) | 308 (4.6%) | 334 (4.8%) |
| Peripheral vascular disease, n (%) | 264 (1.6%) | 108 (1.6%) | 100 (1.5%) | 95 (1.4%) | 99 (1.5%) | 111 (1.6%) | 110 (1.6%) |
| Hypertension, n (%) | 9,476 (56.7%) | 4,004 (57.5%) | 3,844 (56.0%) | 3,681 (55.5%) | 3,836 (56.3%) | 3,901 (57.6%) | 3,901 (56.5%) |
| Dyslipidaemia, n (%) | 8,725 (52.2%) | 3,644 (52.3%) | 3,532 (51.4%) | 3,471 (52.4%) | 3,550 (52.1%) | 3,611 (53.4%) | 3,527 (51.1%) |
| Chronic kidney disease, n (%) | 1,656 (9.9%) | 748 (10.7%) | 722 (10.5%) | 654 (9.9%) | 688 (10.1%) | 683 (10.1%) | 710 (10.3%) |
| Chronic lung disease, n (%) | 1,588 (9.5%) | 692 (9.9%) | 629 (9.2%) | 627 (9.5%) | 714 (10.5%) | 659 (9.7%) | 691 (10.0%) |
| Cancer, n (%) | 1,339 (8.0%) | 551 (7.9%) | 537 (7.8%) | 517 (7.8%) | 526 (7.7%) | 516 (7.6%) | 550 (8.0%) |
| Immunodeficiency, n (%) | 986 (5.9%) | 425 (6.1%) | 432 (6.3%) | 397 (6.0%) | 426 (6.3%) | 448 (6.6%) | 405 (5.9%) |
| HbA1c, mmol/mol (IQR)* | 8,351 (18.4%) | 973 (19.4%) | 996 (19.7%) | 970 (19.2%) | 935 (18.4%) | 894 (17.3%) | 918 (18.3%) |
| eGFR, mL/min/1/. 73m^2^ (IQR)** | 4,421 (9.7%) | 491 (9.8%) | 476 (9.4%) | 492 (9.8%) | 502 (9.9%) | 512 (9.9%) | 473 (9.4%) |
| Antithrombotic medication, n (%) | 3,975 (23.8%) | 1,614 (23.2%) | 1,564 (22.8%) | 1,569 (23.7%) | 1,630 (23.9%) | 1,618 (23.9%) | 1,658 (24.0%) |
| Type 1 diabetes mellitus, n (%) | 7,465 (44.7%) | 3,055 (43.9%) | 3,085 (44.9%) | 2,886 (43.5%) | 3,012 (44.2%) | 3,027 (44.7%) | 3,080 (44.6%) |
| Diabetes duration, median years (IQR) | 9.9 (4.4, 15.2) | 9.8 (4.3, 15.1) | 10.0 (4.4, 15.1) | 9.8 (4.4, 15.1) | 10.0 (4.3, 15.2) | 10.1 (4.3, 15.6) | 10.1 (4.5, 15.2) |
| Insulin treatment, n (%) | 8,554 (51.2%) | 3,574 (51.3%) | 3,524 (51.3%) | 3,370 (50.8%) | 3,489 (51.2%) | 3,467 (51.2%) | 3,518 (51.0%) |
| GLP-1 RA or SGLT2i treatment, n (%) | 6,824 (40.8%) | 2,899 (41.6%) | 2,754 (40.1%) | 2,718 (41.0%) | 2,785 (40.9%) | 2,755 (40.7%) | 2,835 (41.1%) |
| Number of antidiabetic drugs: |  |  |  |  |  |  |  |
| No glucose-lowering medication, n (%) | 1,171 (%) | 543 (7.8%) | 489 (7.1%) | 465 (7.0%) | 526 (7.7%) | 499 (7.4%) | 510 (7.4%) |
| 1 glucose-lowering medications, n (%) | 9,160 (54.8%) | 3,689 (53.0%) | 3,767 (54.8%) | 3,620 (54.6%) | 3,719 (54.6%) | 3,707 (54.8%) | 3,755 (54.4%) |
| 2 glucose-lowering medications, n (%) | 3,815 (22.8%) | 1,589 (22.8%) | 1,556 (22.7%) | 1,549 (23.4%) | 1,532 (22.5%) | 1,524 (22.5%) | 1,526 (22.1%) |
| ≥3 glucose-lowering medications, n (%) | 2,573 (15.4%) | 1,142 (16.4%) | 1,059 (15.4%) | 995 (15.0%) | 1,037 (15.2%) | 1,038 (15.3%) | 1,111 (16.1%) |
